# In Operando Angle‐Resolved Photoemission Spectroscopy with Nanoscale Spatial Resolution: Spatial Mapping of the Electronic Structure of Twisted Bilayer Graphene

**DOI:** 10.1002/smsc.202000075

**Published:** 2021-03-30

**Authors:** Paulina Majchrzak, Ryan Muzzio, Alfred J. H. Jones, Davide Curcio, Klara Volckaert, Deepnarayan Biswas, Jacob Gobbo, Simranjeet Singh, Jeremy T. Robinson, Kenji Watanabe, Takashi Taniguchi, Timur K. Kim, Cephise Cacho, Jill A. Miwa, Philip Hofmann, Jyoti Katoch, Søren Ulstrup

**Affiliations:** ^1^ Department of Physics and Astronomy Aarhus University 8000 Aarhus C Denmark; ^2^ Department of Physics Carnegie Mellon University Pittsburgh, Pennsylvania 15213 USA; ^3^ Electronics Science and Technology Division US Naval Research Laboratory Washington D.C 20375 USA; ^4^ Research Center for Functional Materials National Institute for Materials Science 1-1 Namiki Tsukuba 305-0044 Japan; ^5^ International Center for Materials Nanoarchitectonics National Institute for Materials Science 1-1 Namiki Tsukuba 305-0044 Japan; ^6^ Diamond Light Source Division of Science Didcot United Kingdom

**Keywords:** 2D material devices, angle-resolved photoemission spectroscopy with nanoscale spatial resolution, electron transport, twisted bilayer graphene, van der Waals heterostructures

## Abstract

To pinpoint the electronic and structural mechanisms that affect intrinsic and extrinsic performance limits of 2D material devices, it is of critical importance to resolve the electronic properties on the mesoscopic length scale of such devices under operating conditions. Herein, angle‐resolved photoemission spectroscopy with nanoscale spatial resolution (nanoARPES) is used to map the quasiparticle electronic structure of a twisted bilayer graphene device. The dispersion and linewidth of the Dirac cones associated with top and bottom graphene layers are determined as a function of spatial position on the device under both static and operating conditions. The analysis reveals that microscopic rotational domains in the two graphene layers establish a range of twist angles from 9.8° to 12.7°. Application of current and electrostatic gating lead to strong electric fields with peak strengths of 0.75 V/μm at the rotational domain boundaries in the device. These proof‐of‐principle results demonstrate the potential of nanoARPES to link mesoscale structural variations with electronic states in operating device conditions and to disentangle such extrinsic factors from the intrinsic quasiparticle dispersion.

## Graphical abstract

Nanoscale angle‐resolved photoemission spectroscopy is applied to map the electronic structure of a twisted bilayer graphene device during the application of current and an electrostatic gate voltage. Rotational domains are found to strongly affect the measured linewidth of the top and bottom Dirac cones and lead to a spatially inhomogeneous electric field during operation of the device.

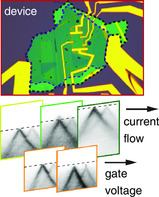

## 1 Introduction

The toolbox of 2D materials encompasses a wide range of electronic states of matter, including metals, superconductors, semiconductors, and insulators, enabling preparation of vertical stacks with varying electrical functionality integrated in the same heterostructure.^[^ ^1^, ^2^, ^3^, ^4^ ^]^ These varied properties can be further augmented via the addition of strain, or formation of a moiré superlattice due to dissimilar lattice constants or twist angles, resulting in new properties from the superposition and interaction of the individual atomic lattices in the stack.^[^ ^2^ ^]^

In twisted bilayer graphene (TBLG), such superlattices can be induced and tuned by varying the interlayer rotation *θ* between two graphene layers. Extensive work has been done to investigate the influence of varied twist angle and doping on the transport and optical properties of TBLG, revealing its potential for versatile applications. When *θ* equals the so‐called magic angle twist of 1.1°, a fascinating temperature‐ and doping‐dependent electronic phase diagram emerges. It bears close resemblance to that of the high‐temperature cuprate superconductors, where an insulating phase is flanked by superconducting domes.^[^ ^5^, ^6^, ^7^ ^]^ The presence of superlattice van Hove singularities in the density of states (DOS) around the Fermi energy suggests that electronic correlations play a key role in establishing this behavior.^[^ ^8^ ^]^ The van Hove singularities are accompanied by a set of flat electronic bands with carrier filling levels that can be controlled with top and bottom gating electrodes, giving access to a large doping range in the TBLG phase diagram.^[^ ^7^, ^9^, ^10^, ^11^ ^]^ Larger twist angles can be utilized to tune the energy difference between the occupied and unoccupied van Hove singularities to be resonant with the wavelength of an optical excitation, leading to an increase in the G‐band intensity in Raman spectroscopy.^[^ ^12^, ^13^, ^14^ ^]^ Asymmetric doping of the TBLG Dirac cones in top and bottom graphene layers causes this optical transition to be indirect, enabling gate‐tunable optical absorption.^[^ ^15^, ^16^, ^17^ ^]^ At a large incommensurate twist angle of 30°, the system forms a quasicrystal with dodecagonal rotational symmetry characterized by mirrored Dirac cones in momentum space.^[^ ^18^, ^19^ ^]^ For an arbitrary large twist angle, the lower graphene layer acts as a buffer layer to screen charge puddles in an underlying silicon dioxide substrate in a device, improving the transport properties of the top graphene layer in the device.^[^ ^20^ ^]^

The sensitivity of the TBLG electronic structure toward small structural variations demands that the electronic and optical properties be probed with nanoscale spatial resolution. Various spectromicroscopic probes have already been used to this end, albeit only indirect measurements that link structural inhomogeneities with electronic structure have been performed thus far. The strong *θ*‐dependence of the optical absorption can be exploited to visualize rotational domains in Raman mapping techniques, which can be combined with low energy electron microscopy^[^ ^13^, ^21^ ^]^ or transmission electron microscopy^[^ ^22^ ^]^ to determine fine rotational smearing and strain variation among microscopic rotational domains in TBLG. Scanning tunneling microscopy and spectroscopy have been utilized to map symmetry‐broken superlattice van Hove singularities on the atomic scale, indicating the existence of nematic order in the superconducting phase of TBLG.^[^ ^23^, ^24^ ^]^ Combinations of transmission electron microscopy and electron transport measurements have shown that atomic reconstructions can lead to separate AA‐, AB‐, and BA‐stacked regions in TBLG, forming topological networks that support helical currents.^[^ ^25^, ^26^, ^27^ ^]^ Nanoscale imaging based on an infrared excitation at the surface, enhanced locally by a metallic tip, can provide maps of the photocurrent across a device and reveal how small variations in *θ* impact optical conductivity.^[^ ^28^ ^]^

Angle‐resolved photoemission spectroscopy (ARPES) with a spatial resolution better than 1 μm, so‐called nanoARPES, is becoming an important complementary tool to the aforementioned mapping capabilities as it provides a means to investigate spatially dependent electronic structure of van der Waals heterostructures.^[^ ^29^, ^30^, ^31^, ^32^, ^33^, ^34^ ^]^ The technique provides access to the position‐ and momentum‐resolved quasiparticle spectral function, which contains information on dispersion and many‐body interactions for low energy excitations around the Fermi level, relevant for electron transport, and toward higher binding energies, relevant for optical transitions.

Furthermore, in situ and in operando noninvasive charge carrier control in the electrostatically gated devices can be achieved in nanoARPES while measuring the dispersion. This capability has only recently been demonstrated in van der Waals heterostructure devices incorporating graphene, bilayer graphene or transition metal dichalcogenides supported on an hBN dielectric and a graphite back gate.^[^ ^35^, ^36^, ^37^, ^38^ ^]^ The approach can be extended to measurements where a current is applied between source and drain electrodes contacted to the heterostructure. So far, such conditions have only been used in an ARPES study on a high temperature superconductor to determine the spectral response to the breakdown current that destroys the coherence of the superconducting quasiparticle,^[^ ^39^, ^40^ ^]^ and in a nanoARPES study that demonstrates the possibility to map the local mobility and impact of defect scattering around structural imperfections in exfoliated graphene on hBN.^[^ ^41^ ^]^ Being able to perform ARPES in the presence of a current density is another major advantage of the nanoscale light spot as the voltage drop across the beam diameter is small, preventing detrimental energy broadening of the measured spectrum.^[^ ^34^, ^41^ ^]^

In this work, we demonstrate the strength of the in operando nanoARPES technique by mapping the Dirac cones in a TBLG device that resolves the microscale twist angle variations. The mapping is carried out in the presence of gate‐induced doping and source–drain currents thereby combining the two standard modes of operation of a device with the nanoARPES capability. We develop an analysis method that is capable of extracting maps composed of the position‐dependent variations in Dirac cone dispersion and linewidth, leading to the identification of rotational domain boundaries and impurities within the device. We show that these features are characterized by significant energy‐ and momentum‐dependent displacements of the Dirac cones when a current is applied, which we attribute to strong local electric field enhancements. Finally, we find that this behavior is also strongly dependent on the electrostatic gate voltage applied to dope the TBLG, thereby revealing a complex interplay of structural inhomogeneity, transport properties and electronic structure using in operando nanoARPES.

## 2 Results and Discussion

### 2.1 Setup of nanoARPES Experiment on a Device

**Figure 1:**
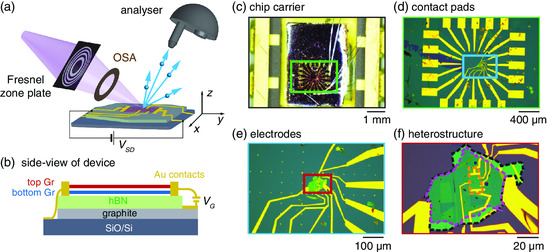
a) Schematic of nanoARPES experiment on TBLG device. A synchrotron beam with an energy of 60 eV is focused onto the device using a Fresnel zone plate and OSA. Photoemitted electrons (blue arrows and spheres) are collected using a hemispherical analyzer. b) Side view schematic of the TBLG heterostructure integrated in a device architecture, permitting source–drain ($V_{\text{SD}}$) and gate ($V_{G}$) voltage tunability in situ. c–f) Optical micrographs presenting the installation of the device in a chip carrier compatible with the nanoARPES experiment and the configuration of electrodes contacted to the heterostructure. Colored boxes demarcate the magnified region shown in the images with correspondingly colored outlines. The TBLG, hBN, and graphite flakes are marked by orange, black, and purple dashed outlines, respectively, in (f).

The primary components of our nanoARPES experiment are shown in **Figure** **1**a. A synchrotron beam with a photon energy of 60 eV is illuminating a Fresnel zone plate. The focused part of the beam is selected by an order sorting aperture (OSA), leading to a spot with a diameter of (690 ± 80) nm on the sample. By scanning the (x,y)‐position of the sample using a piezoelectric manipulator and collecting the angular and energy distributions of photoemitted electrons at each spot with a hemispherical electron analyzer, it is possible to determine the 4D (E,k,x,y)‐dependent photoemission intensity, along a selected direction in the momentum space.

The device we investigate in this study is the same as we used in the study by Jones et al.^[^ ^38^ ^]^ It was prepared using graphene grown on copper foil by chemical vapor deposition (CVD).^[^ ^13^, ^21^, ^42^, ^43^ ^]^ Two graphene flakes were successively transferred onto a hBN dielectric and a graphite back gate, supported on SiO_2_/Si.^[^ ^37^, ^38^, ^44^ ^]^ Source, drain, and gate electrodes connecting to the resulting TBLG flake were defined using several electron beam lithography steps, leading to the device architecture shown in Figures 1b.^[^ ^38^ ^]^ The details of the device are presented via the set of optical micrographs in Figure 1c–f. The SiO_2_/Si wafer with the device was placed in a chip carrier and wire‐bonded (see Figure 1c). Multiple bonding pads, included for the sake of redundancy (see Figure 1d,e), are useful as additional alignment markers for quickly locating the heterostructure and for focusing the beam on a uniform feature with a straight edge during the nanoARPES experiment. Outlines of the TBLG, hBN, and graphite back gate are shown in Figure 1f.

### 2.2 Mapping of Photoemission Intensity

**Figure 2:**
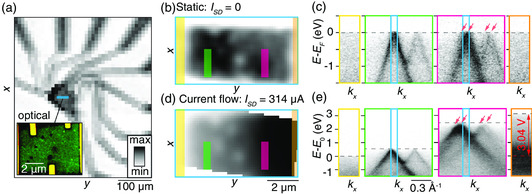
a) Coarse map of the photoemission intensity integrated over the detector. The map is obtained with a scan step size of 10 μm in the *x* and *y* directions, revealing the location of the device when compared with the corresponding optical micrograph in Figure 1e (rotated by 90°). The inset presents a zoomed‐in optical micrograph, which has been digitally enhanced to clearly show the TBLG part of the device. The TBLG corresponds to the “green” contrast. b) Fine photoemission intensity map obtained in static conditions with a scan step size of 250 nm within the region marked by a blue rectangle in (a). c) ARPES spectra spatially integrated over the rectangular regions in (b) with colors of the rectangles corresponding to the outlines of the panels containing the spectra. Arrows indicate four Dirac cones centered at different values of *k*. d,e) Corresponding data for a situation where a current of $I_{\text{SD}}=314$ μA is passed between the source and drain electrodes marked by yellow and orange rectangles in (d). The arrows in the panel with the pink outline in (e) indicate four Dirac cones that shift in both *E* and *k*. The voltage drop across the device is 3.04 V (see double‐headed arrow and dashed lines in the panel with an orange outline in (e)). The energy scale is referenced to the Fermi edge, $E_{0}$, in the left electrode. The photoemission intensity in (b) and (d) is composed from the (E,k)‐integrated intensity within the blue boxes in (c) and (e), respectively.

To locate the region of the device with the TBLG flake, a coarse (x,y)‐scan over an area of 0.47 by 0.40 mm^2^ with a step size of 0.01 mm, covering an appreciable part of the sample, is initially carried out. The resulting map of the photoemission intensity integrated over the detector is shown in **Figure** **2**a, and closely resembles the optical micrograph of the same region of the sample in Figure 1e. Such a coarse map takes 20 min to acquire and thereby provides a quick overview of the entire sample, reassuring that the electrical connections of the flakes and electrodes are intact. If they were damaged, those parts of the device would charge and thereby not display any signal.^[^ ^45^ ^]^ A fine scan over an area of 5.25 by 10.75 μm^2^ with a step size of 0.25 μm, corresponding to the region containing the TBLG flake demarcated by a blue rectangle and shown in the zoomed‐in optical micrograph in the inset in Figure 2a, is shown in Figure 2b. As a high quality ARPES spectrum is required at each position to facilitate a detailed analysis, the acquisition time for such a map is normally on the order of 4 h. Note that, the three small electrodes that are visible on the side of the TBLG device in the optical image in Figure 2a are floated and therefore do not influence the function of the device.

Once the TBLG region has been located, the sample is carefully aligned such that the photoelectrons are measured along the line connecting the Dirac points corresponding to the top and bottom layers, which we define as the $k_{x}$ direction. Representative E(k)‐dispersion plots for different regions on the sample are shown in Figure 2c. These have been integrated over the (x,y)‐regions corresponding to the electrodes and TBLG areas demarcated by colored boxes in Figure 2b. The map in Figure 2b, in turn, is composed from the (E,k)‐integrated intensity within the spectral region around the $\bar{K}$‐point of the top graphene layer, indicated by blue boxes in Figure 2c. The photoemission intensity from the polycrystalline electrodes is uniform with a sharp cut‐off at the Fermi level, *E*_F_. The TBLG regions display two Dirac cones, displaced in *k* due to the twist angle between the graphene layers, with the intensity from the cone in the bottom layer being substantially weaker than from the top layer due to the inelastic attenuation of the photoelectrons. Close to *E*_F_, the Dirac cones appear to behave as in single‐layer graphene, in that they do not interact in this spectral region. Around 1 eV below *E*_F_, the two cones intersect, which leads to a hybridization‐induced minigap and the formation of saddle points, as described in previous ARPES studies of TBLG at similar twist angles.^[^ ^38^, ^46^, ^47^, ^48^, ^49^ ^]^ A substantial increase in the broadening of the spectra and the appearance of additional faint Dirac cones displaced in *k* are noticeable for some positions, as seen via the arrows on the dispersion in the panel with the pink outline in Figure 2c. These variations of the Dirac cone linewidth and position are ultimately responsible for the changes of intensity that are visible in the TBLG area of the map in Figure 2b. In the following sections, we will explore in much more detail what causes these spatially varying features in the intensity.

Figure 2d,e shows corresponding nanoARPES measurements, whereas a current, *I*_SD_, of 314 μA is passed between the source and drain electrodes. Note that no gate voltage is applied here. The color gradient across the device in Figure 2d shows the voltage drop, which rigidly shifts the energy of the ARPES spectra within the (E,k)‐integration window that the map is composed from (see blue boxes in Figure 2e). This shift is clearly visible in Figure 2e where a total voltage drop of 3.04 V is determined using the position of the Fermi edge in the left and right electrodes. The ARPES spectra from the TBLG regions gradually shift in energy along the device, adhering to the local potential *ϕ*. Note that we reference the energy scale to the Fermi edge, $E_{0}$, in the left electrode. Interestingly, the dispersion in Figure 2e from the region marked by a pink box shows two sets of Dirac cones from top and bottom graphene layers that are displaced not only in *k*, but also in *E* (see guiding arrows in the figure). In the following, we introduce a method that gives a precise estimate of the real space location of such features both in static and operating conditions.

### 2.3 Analysis of Spectral Function

**Figure 3:**
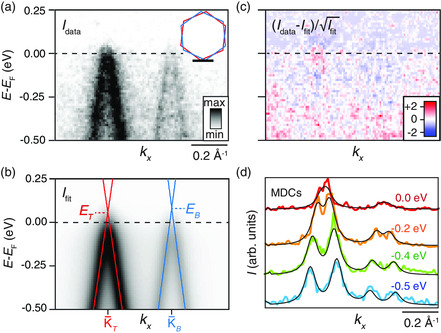
a) Photoemission intensity, $I_{\text{data}}$, from the location with the sharpest Dirac cones in the TBLG. The inset shows a sketch of the BZs for TBLG with the black line indicating our ARPES cut. b) Corresponding fit of the intensity, $I_{\text{fit}}$. Solid lines are the linear bare bands resulting from the fit with top (bottom) Dirac point energy $E_{T(\text{B)}}$ and position ${\bar{K}}_{T(\text{B)}}$. c) Normalized residual plot assuming Poissonian statistics, demonstrating the high quality of the fit. d) Momentum distribution curves (MDCs) extracted from the data (colored) and fit (black) at the given energies.

To extract the spatially varying linewidth and position of the two Dirac cones and to determine the local twist angle in the device, we extend a method to extract these quantities from the photoemission intensity.^[^ ^41^, ^50^ ^]^ **Figure** **3**a shows a detailed ARPES snapshot of a region of the TBLG sample with two sharp Dirac cones. We restrict the following analysis to the noninteracting part of the cones around the (E,k)‐range shown in Figure 3a.

We describe the photoemission intensity from the top (bottom) Dirac cone in static conditions using the expression $I_{T(\text{B)}}(E,k)={\left|M_{T(\text{B)}}\right|}^{2}A_{T(\text{B)}}(E,k)f_{\text{FD}}(E)$. Here, $f_{\text{FD}}$ is the Fermi‐Dirac function, and $M_{T(\text{B)}}$ is the dipole matrix element which is assumed to be constant for each branch within the layers for simplicity. This is a reasonable approximation for our ARPES cut through the two Dirac points. The inset of Figure 3a shows the hexagonal Brillouin zones (BZs) for the upper and lower layers separately, illustrating the origin for the *k*‐displacement of the Dirac points of the upper and lower layers. The intensity in the two branches is nearly symmetric along this cut, which is in sharp contrast to the so‐called “dark corridor,” which arises along the $\bar{\Gamma }-\bar{K}$ line, nearly orthogonal to our cut.^[^ ^41^, ^51^ ^]^ The spectral function of top (bottom) Dirac cone, $A_{T(\text{B)}}$, is described by

(1) $$A_{T(\text{B)}}(E,k)=\frac{{\left(2\pi \right)}^{-1}W_{T(\text{B)}}\hbar v_{T(\text{B)}}}{{\left(E-\hbar v_{T(\text{B)}}|k+\Delta K_{T(\text{B)}}|-E_{T(\text{B)}}\right)}^{2}+{\left(W_{T(\text{B)}}\hbar v_{T(\text{B)}}/2\right)}^{2}}$$

where $E_{T(\text{B)}}$ is the top (bottom) Dirac point energy, $\Delta K_{T(\text{B)}}$ is a rigid *k* shift relative to the top (bottom) Dirac point position ${\bar{K}}_{T(\text{B)}}$, shown in Figure 3b, and $W_{T(\text{B)}}$ is the linewidth of top (bottom) momentum distribution curves (MDCs). The top (bottom) band velocity, $v_{T(\text{B)}}$, that defines the slopes of the linear branches can be described by the fixed value $v_{T}=1.10\times 10^{6}$ ($v_{B}=1.21\times 10^{6}$) m s^−1^ that we found for the same device studied in the work by Jones et al.^[^ ^38^ ^]^

In this scheme, the broadening given by $W_{T(\text{B)}}$ is independent of energy and momentum. It does not distinguish between intrinsic linewidth broadening due to quasiparticle scattering involving defects and extrinsic broadening mechanisms that lead to juxtaposition of multiple Dirac cones shifted in *E* and/or *k*. This description turns out to be sufficient for understanding the main features of our device, but the model can be extended to disentangle these different contributions.

The total photoemission intensity is calculated as $I_{\text{tot}}=I_{T}+I_{B}+I_{0}$, where $I_{0}$ is a linear background contribution, and finally convoluted with Gaussian functions to account for the energy and momentum resolution of the experiment. The result of fitting this model to the ARPES intensity in Figure 3a is seen in the image in Figure 3b. The bare dispersions are obtained along with the Dirac point energies $E_{T}=(60\pm 10)$ meV and $E_{B}=(110\pm 40)$ meV. Note that the error bars provided here pertain to the fit and do not reflect the overall accuracy of determining the Dirac point energies, as these are affected by the precise alignment of the momentum space cut. We will return to this issue in the following discussion. The fitted energies reflect a hole doping effect that may be caused by residual water on the surface or polymer residues in the heterostack. The quality of the fit is monitored through the normalized residual, which is shown in Figure 3c. Assuming that the noise can be described by Poissonian statistics, 95% of the normalized residual must be within the range of ±2 for the fit to be acceptable. The agreement of the model with the data is further demonstrated via the MDCs extracted from the data and the fit shown in Figure 3d.

### 2.4 Spectroscopic Fingerprints of Rotational Domain Boundaries

Applying the spectral function analysis to the (E,k,x,y)‐dependent nanoARPES intensity of the full TBLG flake between source and drain electrodes in Figure 2b enables us to probe the spatially dependent linewidths and *k* shifts of the two Dirac cones as well as the *k* separation, ΔK, between them. We also obtain the local doping via $E_{T(\text{B)}}$, however, we will discuss this in detail later in connection with electrostatic gating of the device.

**Figure 4:**
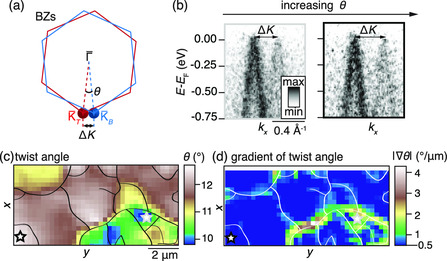
a) Schematic of BZs for the two graphene flakes in the TBLG, relating the twist angle, *θ*, to the *k* separation, ΔK, between top and bottom Dirac points indicated by red and blue spheres, respectively. b) ARPES snapshots from areas with twist angles of 9.8° and 12.7°. Double‐headed arrows mark the separation of the Dirac cones. c) Spatial map of *θ* extracted from spectral function analysis. Gray and black stars indicate the areas from which the snapshots in (b) are extracted. d) Corresponding gradient of *θ*. The outlines in (c) and (d) indicate the center of regions where |∇θ|≥0.5° μm^−1^.

The extracted values of ΔK can be converted to estimates of the local twist angle using the relation $\theta =2\arcsin(\Delta K/2|\bar{K}|)$, which follows from the sketch of the TBLG BZs in **Figure** **4**a. The method of extracting ΔK from single (E,k) cuts, shown for the extreme values of *θ* in Figure 4b, leads to an error bar on the values of *θ* of 0.2° for the results presented here. This is caused by the possibility of cutting slightly off ${\bar{K}}_{T}$ and ${\bar{K}}_{B}$, as the Dirac cones may shift in both $k_{x}$ and $k_{y}$ directions in a given (x,y) position. A complete $\left(E,k_{x},k_{y}\right)$ scan for each position would provide a more precise value of ΔK, however, such a 5D data set would not be feasible to collect with the low photon flux provided by the zone plate in this nanoARPES experiment.

The resulting map of *θ* in Figure 4c shows a variation from 9.8° to 12.7° within the device. We observe regions separated by abrupt changes of *θ*, as indicated by a high value of the gradient |∇θ| in Figure 4d. We choose a value of 0.5° μm^−1^ as a lower bound that indicates a significant local twist angle change in the map. Outlines have been drawn through the center of regions where |∇θ|≥0.5° μm^−1^ as a guide to the eye in Figure 4c,d. These regions are boundaries that delineate microscopic rotational domains in the TBLG structure. The length scale of these domains is on the order of 2 μm, which agrees well with Raman spectroscopy and electron diffraction studies of similar CVD TBLG samples.^[^ ^13^, ^21^ ^]^ The value of *θ* also varies continuously within these domains, suggesting minute angle rotations of the flakes on a length scale that is smaller than we can resolve with the 690 nm beam.

**Figure 5:**
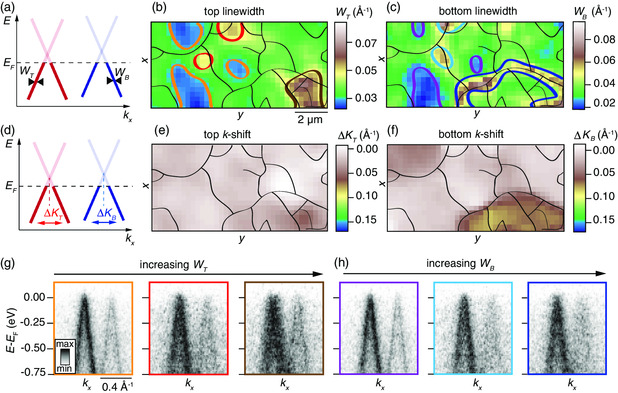
a) Sketch of top and bottom Dirac cones with indication of MDC linewidth parameters $W_{T}$ and $W_{B}$. b,c) Linewidths of b) top and c) bottom Dirac cones extracted from photoemission intensity fits. d) Sketch of Dirac cones with indication of *k*‐shift parameters $\Delta K_{T}$ and $\Delta K_{B}$ for top and bottom Dirac cones, respectively. e,f) Extracted maps of *k* shifts for e) top and f) bottom Dirac cones. The black outlines from Figure 4c are shown as a guide to the eye in (b,c,e,f). The color scales in these maps are kept identical with the corresponding panels in Figure 7 to enable a direct comparison of the maps. g,h) ARPES spectra for increasing linewidth in g) top and h) bottom Dirac cones. The (E,k)‐dependent intensity represents a spatial average of the regions demarcated by outlines in (b,c) with the same color coding between panels and outlines.

The fits of the photoemission intensity yield the energy‐independent linewidth of the top (bottom) Dirac cone given by $W_{T}$ ($W_{B}$), as shown in **Figure** **5**a, which are shown as a function of (x,y) position in Figure 5b,c. The (x,y)‐dependent shift of $\Delta K_{T}$ ($\Delta K_{B}$) of the top (bottom) Dirac cone, shown in Figure 5d, is simultaneously determined, as shown in the maps in Figure 5e,f. All maps represent the same area, as shown in Figure 4c,d, and the same outlines of rotational domain boundaries have been overlaid as a guide to the eye. Colored outlines have been added in the maps of $W_{T}$ and $W_{B}$ around (x,y) regions with extreme linewidth values. These changes are easily seen in the spatially averaged E(k) dispersions for these regions, as shown in Figure 5g,h.

The orange and purple outlines in the maps of $W_{T}$ and $W_{B}$, respectively, correspond to areas with the sharpest Dirac cones in both layers, indicating especially high quality regions within the device. Indeed, these regions are observed to be centered within rotational domains by comparing the location of these colored outlines with the black outlines. Red and light blue outlines for $W_{T}$ and $W_{B}$, respectively, demarcate areas that are characterized by Dirac cones that are a factor of 2.2 broader than in the optimal parts within the domains. These areas appear rather localized and overlap between the top and bottom flakes. The simultaneous broadening of both top and bottom Dirac cones indicates that these areas contain impurities that are likely to be encapsulated between the hBN and the TBLG, thereby affecting both graphene flakes. Here, we refer to impurities in a very broad sense that includes wrinkles, bubbles, trapped water, or trapped residues from the transfer processes.^[^ ^52^ ^]^ We also identify areas where the top Dirac cone is broad whereas the bottom Dirac cone is rather sharp, as shown via the brown outline in Figure 5b, and the corresponding spectrum in Figure 5g. In Figure 5e, a large $K_{T}$ shift is visible in the lower right corner, which is the same area, as shown by a brown outline in Figure 5b. The opposite situation occurs in the region with the dark‐blue outline in Figure 5c, as shown in the related spectrum in Figure 5h. A large shift of $K_{B}$ is visible in Figure 5f around the area indicated by the dark‐blue outline in Figure 5c. This behavior is linked to the different rotational domains present within the two graphene layers. When these rotational domains are superimposed, they give rise to the spatial dependence of *θ* shown in Figure 4c and explain the spatial linewidth and *k* shift variations reported in the E(k) dispersions.

### 2.5 Local Electronic Structure of the Device in Operating Conditions

**Figure 6:**
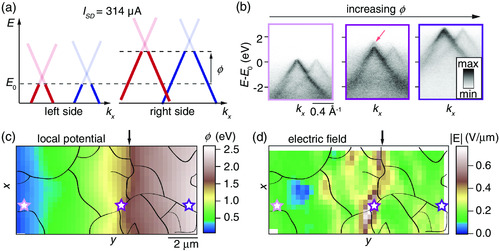
a) Schematic of rigid energy shift of the Dirac cones measured by ARPES when a current is applied between source and drain electrodes. The energy shift indicates the local potential *ϕ*, which is referenced to the Fermi energy, $E_{0}$, in the left electrode. b) ARPES spectra obtained from the regions marked by colored stars in (c) with the color coding following the panel outlines. c) Map of *ϕ* determined via (E,k)‐dependent fits of the intensity for every (x,y) position. d) Corresponding electric field, |E|=|∇ϕ|. The arrow indicates a rotational domain boundary along *x* with a significant increase in the local field.

The effect of rotational domain boundaries in the presence of a current of 314 μA is investigated in **Figure** **6**. As mentioned previously in the discussion of Figure 2, and as shown in Figure 6a, the main effect of a current is a position‐dependent rigid energy shift of the spectra caused by the local potential *ϕ*. The effect is clearly illustrated by the measured spectra from the left, middle, and right sides of the device in Figure 6b. By considering these rigid energy shifts in the fits of the nanoARPES data represented by the map in Figure 2d, we are able to extract a map of the local potential, which is shown with the rotational domain boundaries superimposed in Figure 6c. Subtle details in *ϕ* are difficult to identify from such a map, because the overall voltage drop across the device is the dominating effect. It is instead more instructive to calculate the position‐dependent electric field strength |E|=|∇ϕ|, as shown in Figure 6d. Interestingly, in the middle of the device, where a rotational domain boundary is seen to perforate the TBLG from top to bottom edges, the electric field exhibits a substantial increase, reaching a maximum strength of 0.75 V μm^−1^. This abrupt change is directly visible in the corresponding ARPES spectrum from this region in Figure 6b where a faint replica of the top Dirac cone, rigidly shifted in energy by 0.44 eV, is observed (see arrow in the middle panel of Figure 6b). This effect arises because the light spot is large enough to illuminate the sharp boundaries between two regions with different local potential, leading to the incoherent superposition of the intensity from the two sides of the boundary. The potential change in this region provides an estimate for the rotational domain boundary resistance, which we calculate to be 9.6 kΩμm.

**Figure 7:**
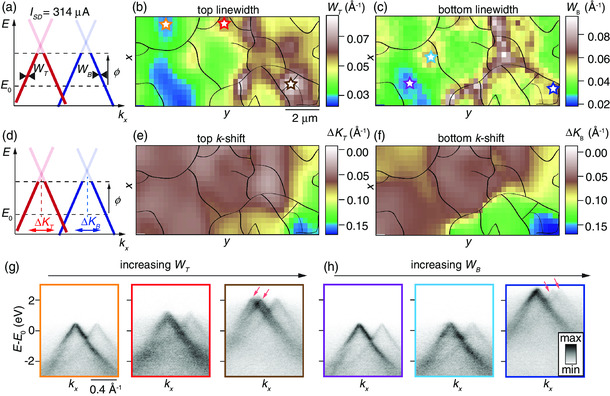
a–f) Linewidth and *k*‐shift maps resulting from spectral function analysis, similar to Figure 5a–f, but for $I_{\text{SD}}=314$ μA. The color scales are identical to those in the corresponding maps in Figure 5b,c,e,f. g,h) ARPES spectra obtained from areas indicated by colored stars in (b,c). Stars in the maps of (b) and (c) and panel outlines in (g) and (h) are linked via the color coding. Arrows in (g) and (h) indicate Dirac cones shifted in *E* and *k*. Black lines overlaid on the maps in (b,c,e,f) delineate rotational domain boundaries determined from the analysis in Figure 4c,d.

The associated (x,y)‐dependent linewidths and *k* shifts of the Dirac cones in the presence of current are shown in **Figure** **7**a–f, which repeat the analysis carried out in static conditions in Figure 5a–f. Note also that the color scales in the maps are identical between the figures to facilitate a direct comparison between the results. Example spectra are shown in Figure 7g,h from the areas marked by stars in Figure 7b,c, with the same color coding. The panels exhibit, from left to right, the dispersion from a high quality region, an impurity, and a domain boundary. Spectra from rotational domain boundaries exhibit complex features that can be described as multiple Dirac cones shifted in both *E* and *k*, as shown via arrows in Figure 7g,h. The shifts are substantially larger than what is observed in the map under static conditions and are explained by the presence of an additional spatially dependent electric field shown in Figure 6d. As our spectral function analysis does not incorporate multiple cones, these features are compensated for in the fit by increasing the values of $W_{T}$ and $W_{B}$. The resulting large values of $W_{T}$ and $W_{B}$, which are pinned to the rotational domain boundary outlines in Figure 7b,c, are therefore an indication for the behavior of the electric field around these defects (see Figure 6d). The observed local spikes in the electric field strength are associated with a spatially dependent increase in resistivity, as shown for a single‐layer graphene device.^[^ ^41^ ^]^

**Figure 8:**
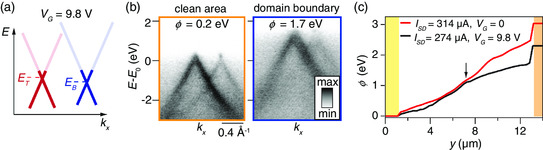
a) Schematic of the effect of electrostatic doping on the Dirac point energies $E_{T}$ and $E_{B}$. b) In operando ARPES spectra from the areas marked by orange and dark‐blue stars in Figure 7b,c ($I_{\text{SD}}=274$ μA and $V_{G}=9.8$ V). c) Profiles of the local potential for the given device operating conditions. The arrow indicates the location of a rotational domain boundary that perforates the whole device along the *x* direction, which is also marked by arrows in Figure 6c,d. The yellow and orange regions indicate the electrodes.

The influence of *n*‐doping on the local potential and the ARPES spectra is investigated by applying an electrostatic gate voltage given by $V_{G}=9.8$ V simultaneously with a current of 274 μA. The current is kept slightly lower with a finite *V*_G_, as we observed a gate leakage current that would tend to critically rise for higher currents. **Figure** **8**a shows the gate‐induced *n*‐doping, i.e., a shift of the Dirac point energy below *E*_F_. Example spectra are shown in Figure 8b for a clean area in the left part of the device and for a rotational domain boundary in the right sides of device. The spectra have been obtained from the areas indicated by orange and dark‐blue stars in Figure 7b,c. The Dirac point region is visible in the bottom Dirac cone in the clean area, indicating a significant *n*‐doping effect. This is less obvious in the top Dirac cone because of a smaller achievable doping with the bottom gate electrode due to screening of charges by the bottom graphene layer.^[^ ^38^ ^]^ Our fits of the photoemission intensity provide a simple estimate of Dirac point energies, $E_{T}$ and $E_{B}$, and reveal that the doping in the top layer varies over the range $(1-3)\times 10^{12}$ cm^−2^ while the doping in the bottom layer varies over the range $(2-7)\times 10^{12}$ cm^−2^ within the device. Such a wide margin for the doping may indicate an inhomogeneous doping, associated with impurities and domain boundaries, which are poorly electrically connected (see spectrum from rotational domain boundary in Figure 8b). However, there is also the issue that the Dirac cone can exhibit position‐dependent shifts not only in $k_{x}$, i.e., along the analyzer slit direction, but also in $k_{y}$. Such shifts can be further enhanced by the presence of electric fields from the finite gate and source–drain voltages that would distort the photoelectron trajectories. Thus, we could be measuring the photoemission intensity from a cut slightly displaced from the vertex of the Dirac point, leading to a position‐dependent overestimation of the doping. Furthermore, due to a nonlinear shape of the Dirac point region in ARPES spectra, a better estimate of doping would require scanning the full Fermi surface of each Dirac cone for every position in the device.^[^ ^37^ ^]^ This would again require a 5D nanoARPES data set, which is not feasible in this experiment. The actual doping will be somewhat smaller than the estimates given here.

The local potential along the device is shown for the situations with and without a finite *V*_G_ in Figure 8c. When $V_{G}=0$, the graphene layers are more resistive because of the smaller doping. The combination of higher current and resistance leads to *ϕ* generally being higher in this situation. The step‐like shift of *ϕ* of 0.22 (0.74) eV at the left (right) electrode boundary reflects the contact resistance, which is calculated to be 4.8 (16.9) kΩμm for $V_{G}=0$ and $I_{\text{SD}}=314$ μA. We point out that there are rotational domain boundaries close to the right electrode (see Figure 4, 5, 6, 7) where the contact resistance is observed to be largest. The potential behaves similar for the two settings of $V_{G}$ in the left side of the TBLG part of the device until about y=7 μm (see arrow in Figure 8c) where it dramatically branches toward the right side. This behavior sets in at the same location marked by an arrow in Figure 6c,d, where rotational domain boundaries perforate from top to bottom edges of the device, giving rise to a high local electric field. This nonlinear behavior of the potential corresponds to a complex spatial dependence of the resistivity of the device caused by the rotational domain boundaries. This spatial dependence is additionally modulated when a finite gate voltage is applied, leading to a highly complex resistivity function $\rho (x,y,V_{G},V_{\text{SD}})$. It is critical that the structural defects that give rise to this complex function can be disentangled from the intrinsic transport properties of TBLG around $E_{F}$ when interpreting low‐energy phenomena, including the temperature‐dependence of *ρ*, electron‐lattice interactions and thermal conductivity.^[^ ^22^, ^53^, ^54^ ^]^ Using nanoARPES, we can separate regions based on the quality of the local electronic structure, as shown above, and access these properties in both pristine and defective parts of a device. We note that the synchrotron beam will additionally induce a photocurrent which must be dissipated in the device, and this could further complicate the overall behavior of the local potential.

## 3 Conclusion

In summary, we have introduced the capability to map Dirac cone linewidths and dispersion in a TBLG device using nanoARPES in combination with several operating modes of the device. In static conditions, our analysis reveals the presence of microscopic rotational domains on a length scale of 2 μm with a range of twist angles from 9.8° to 12.7°. The Dirac cones of the TBLG are observed to exhibit substantial shifts in energy and momentum around the rotational domain boundaries in the presence of a finite current and electrostatic doping, caused by high local electric fields and thereby a complex spatial dependence of the resistivity. We believe that there is a potential for making groundbreaking discoveries of how quantum states evolve under realistic operating conditions with the capability of applying a current and electrostatically doping 2D material devices while measuring their energy‐ and momentum‐dependent spectral function. In particular, advanced 2D heterostructures that combine materials with variable electron‐ and hole‐type doping along the device, tailored 1D conduction channels, or even combinations of dissimilar electrical properties such as semiconductors and superconductors, will display complex transport properties that emerge from the local electronic structure, which can be uncovered by in operando nanoARPES.

## 4 Experimental Section

4.1

4.1.1

##### Sample preparation

The graphene flakes were initially grown on 25 μm‐thick copper foil by CVD using H_2_ and CH_4_ gases following well‐established methods.^[^ ^13^, ^21^, ^42^, ^43^ ^]^ Two CVD graphene flakes were successively transferred onto prestacked 30 nm‐thick hBN on 10 nm‐thick graphite, supported on a SiO_2_ (300 nm)/Si wafer with predefined pads for wire bonding and large electrodes.^[^ ^37^, ^38^, ^44^ ^]^ The initial stacking of hBN and graphite was carried out using a custom‐built transfer tool. Using a thin polycarbonate (PC) film on top of poly(dimethylsiloxane) (PDMS), the hBN was first picked up from a SiO_2_/Si substrate and then used to pick up the graphite flake.^[^ ^30^ ^]^ The stack was then dropped on the SiO_2_/Si wafer. Polymer residues were removed by annealing the stack at 623 K for 15 min in high vacuum. The transferred TBLG flake on hBN/graphite was etched into a narrow stripe. Source, drain and gate electrodes were defined using several electron beam lithography steps. The device was placed in CSB00815 chip package and wire‐bonded to the nanoARPES sample holder and finally annealed in the ultrahigh vacuum system connected to the nanoARPES analysis chamber at 420 K for 90 min before the measurements. The device used in this study was the same as used in the study by Jones et al.^[^ ^38^ ^]^

##### NanoARPES Experiments

The nanoARPES measurements were carried out at the I05 beamline of Diamond Light Source. A spot‐size of (690 ± 80) nm was achieved on the sample by focusing the 60 eV synchrotron beam using a Fresnel zone plate in combination with an OSA. The (E,k,x,y)‐dependent photoemission intensity was obtained by scanning the sample position using a piezoelectric manipulator and collecting the angular and energy distributions of photoemitted electrons at each spot with a Scienta Omicron DA30 hemispherical electron analyzer. The energy and angular resolution were set to 30 meV and 0.2°, limited by the requirement to achieve a reasonable signal‐to‐noise ratio with the low photon flux of $10^{10}$ photons/s caused by the use of a zone plate. The sample was held at a temperature of 70 K during the measurements.

## Conflict of Interest

The authors declare no conflict of interest.

## Data Availability Statement

The data that support the findings of this study are available from the corresponding author upon reasonable request.

## References

[1] K. S. Novoselov , D. Jiang , F. Schedin , T. J. Booth , V. V. Khotkevich , S. V. Morozov , A. K. Geim , Proc. Natl. Acad. Sci. 2005, 102, 10451. 16027370 10.1073/pnas.0502848102 PMC1180777

[2] A. Geim , I. Grigorieva , Nature 2013, 499, 419. 23887427 10.1038/nature12385

[3] Y. Liu , S. Zhang , J. He , Z. M. Wang , Z. Liu , Nano-Micro Lett. 2019, 11, 13. 10.1007/s40820-019-0245-5 PMC7770868 34137973

[4] S. Fan , Q. A. Vu , M. D. Tran , S. Adhikari , Y. H. Lee , 2D Mater. 2020, 7, 022005.

[5] Y. Cao , V. Fatemi , S. Fang , K. Watanabe , T. Taniguchi , E. Kaxiras , P. Jarillo-Herrero , Nature 2018, 556, 43. 29512651 10.1038/nature26160

[6] Y. Cao , V. Fatemi , A. Demir , S. Fang , S. L. Tomarken , J. Y. Luo , J. D. Sanchez-Yamagishi , K. Watanabe , T. Taniguchi , E. Kaxiras , R. C. Ashoori , P. Jarillo-Herrero , Nature 2018, 556, 80. 29512654 10.1038/nature26154

[7] E. Y. Andrei , A. H. MacDonald , Nat. Mater. 2020, 19, 1265. 33208935 10.1038/s41563-020-00840-0

[8] G. Li , A. Luican , J. M. B. Lopes dos Santos , A. H. Castro Neto , A. Reina , J. Kong , E. Y. Andrei , Nat. Phys. 2010, 6, 109.

[9] R. Bistritzer , A. H. MacDonald , Proc. Natl. Acad. Sci. 2011, 108, 12233. 21730173 10.1073/pnas.1108174108 PMC3145708

[10] J. D. Sanchez-Yamagishi , T. Taychatanapat , K. Watanabe , T. Taniguchi , A. Yacoby , P. Jarillo-Herrero , Phys. Rev. Lett. 2012, 108, 076601. 22401231 10.1103/PhysRevLett.108.076601

[11] Y. Cao , J. Y. Luo , V. Fatemi , S. Fang , J. D. Sanchez-Yamagishi , K. Watanabe , T. Taniguchi , E. Kaxiras , P. Jarillo-Herrero , Phys. Rev. Lett. 2016, 117, 116804. 27661712 10.1103/PhysRevLett.117.116804

[12] R. W. Havener , H. Zhuang , L. Brown , R. G. Hennig , J. Park , Nano Lett. 2012, 12, 3162. 22612855 10.1021/nl301137k

[13] J. T. Robinson , S. W. Schmucker , C. B. Diaconescu , J. P. Long , J. C. Culbertson , T. Ohta , A. L. Friedman , T. E. Beechem , ACS Nano 2013, 7, 637. 23240977 10.1021/nn304834p

[14] R. W. Havener , Y. Liang , L. Brown , L. Yang , J. Park , Nano Lett. 2014, 14, 3353. 24798502 10.1021/nl500823k

[15] J. M. B. Lopes dos Santos , N. M. R. Peres , A. H. Castro Neto , Phys. Rev. Lett. 2007, 99, 256802. 18233543 10.1103/PhysRevLett.99.256802

[16] C.-H. Yeh , Y.-C. Lin , Y.-C. Chen , C.-C. Lu , Z. Liu , K. Suenaga , P.-W. Chiu , ACS Nano 2014, 8, 6962. 24999754 10.1021/nn501775h

[17] T.-F. Chung , R. He , T.-L. Wu , Y. P. Chen , Nano Lett. 2015, 15, 1203. 25621859 10.1021/nl504318a

[18] W. Yao , E. Wang , C. Bao , Y. Zhang , K. Zhang , K. Bao , C. K. Chan , C. Chen , J. Avila , M. C. Asensio , J. Zhu , S. Zhou , Proc. Natl. Acad. Sci. 2018, 115, 6928. 29915054 10.1073/pnas.1720865115 PMC6142217

[19] P. Moon , M. Koshino , Y.-W. Son , Phys. Rev. B 2019, 99, 165430.

[20] C.-P. Lu , M. Rodriguez-Vega , G. Li , A. Luican-Mayer , K. Watanabe , T. Taniguchi , E. Rossi , and E. Y. Andrei , Proc. Natl. Acad. Sci. 2016, 113, 6623. 27302949 10.1073/pnas.1606278113 PMC4914180

[21] T. E. Beechem , T. Ohta , B. Diaconescu , J. T. Robinson , ACS Nano 2014, 8, 1655. 24460413 10.1021/nn405999z

[22] T. B. Limbu , K. R. Hahn , F. Mendoza , S. Sahoo , J. J. Razink , R. S. Katiyar , B. R. Weiner , G. Morell , Carbon 2017, 117, 367.

[23] A. Kerelsky , L. J. McGilly , D. M. Kennes , L. Xian , M. Yankowitz , S. Chen , K. Watanabe , T. Taniguchi , J. Hone , C. Dean , A. Rubio , A. N. Pasupathy , Nature 2019, 572, 95. 31367030 10.1038/s41586-019-1431-9

[24] Y. Xie , B. Lian , B. Jack , X. Liu , C.-L. Chiu , K. Watanabe , T. Taniguchi , B. A. Bernevig , A. Yazdani , Nature 2019, 572, 101. 31367031 10.1038/s41586-019-1422-x

[25] S. Huang , K. Kim , D. K. E_mkin , T. Lovorn , T. Taniguchi , K. Watanabe , A. H. MacDonald , E. Tutuc , B. J. LeRoy , Phys. Rev. Lett. 2018, 121, 037702. 30085814 10.1103/PhysRevLett.121.037702

[26] P. Rickhaus , J. Wallbank , S. Slizovskiy , R. Pisoni , H. Overweg , Y. Lee , M. Eich , M.-H. Liu , K. Watanabe , T. Taniguchi , T. Ihn , K. Ensslin , Nano Lett. 2018, 18, 6725. 30336041 10.1021/acs.nanolett.8b02387

[27] H. Yoo , R. Engelke , S. Carr , S. Fang , K. Zhang , P. Cazeaux , S. H. Sung , R. Hovden , A. W. Tsen , T. Taniguchi , K. Watanabe , G.-C. Yi , M. Kim , M. Luskin , E. B. Tadmor , E. Kaxiras , P. Kim , Nat. Mater. 2019, 18, 448. 30988451 10.1038/s41563-019-0346-z

[28] S. S. Sunku , A. S. McLeod , T. Stauber , H. Yoo , D. Halbertal , G. Ni , A. Sternbach , B.-Y. Jiang , T. Taniguchi , K. Watanabe , P. Kim , M. M. Fogler , D. N. Basov , Nano Lett. 2020, 20, 2958. 32052976 10.1021/acs.nanolett.9b04637

[29] N. R. Wilson , P. V. Nguyen , K. Seyler , P. Rivera , A. J. Marsden , Z. P. L. Laker , G. C. Constantinescu , V. Kandyba , A. Barinov , N. D. M. Hine , X. Xu , D. H. Cobden , Sci. Adv. 2017, 3, e1601832. 28246636 10.1126/sciadv.1601832 PMC5298850

[30] J. Katoch , S. Ulstrup , R. J. Koch , S. Moser , K. M. McCreary , S. Singh , J. Xu , B. T. Jonker , R. K. Kawakami , A. Bostwick , E. Rotenberg , C. Jozwiak , Nat. Phys. 2018, 14, 355.

[31] S. Ulstrup , R. J. Koch , S. Singh , K. M. McCreary , B. T. Jonker , J. T. Robinson , C. Jozwiak , E. Rotenberg , A. Bostwick , J. Katoch , J. A. Miwa , Sci. Adv. 2020, 6, eaay6104. 32284971 10.1126/sciadv.aay6104 PMC7124957

[32] S. Ulstrup , C. E. Giusca , J. A. Miwa , C. E. Sanders , A. Browning , P. Dudin , C. Cacho , O. Kazakova , D. K. Gaskill , R. L. Myers-Ward , T. Zhang , M. Terrones , P. Hofmann , Nat. Commun., 2019, 10, 3283. 31337765 10.1038/s41467-019-11253-2 PMC6650412

[33] F. Joucken , E. A. Quezada-Lopez , J. Avila , C. Chen , J. L. Davenport , H. Chen , K. Watanabe , T. Taniguchi , M. C. Asensio , J. Velasco , Phys. Rev. B 2019, 99, 161406.

[34] P. Hofmann , In operando devices studied by angle-resolved photoemission spectroscopy, 2020, arXiv:2011.11490 [cond-mat.str-el].

[35] F. Joucken , J. Avila , Z. Ge , E. A. Quezada-Lopez , H. Yi , R. Le Goff , E. Baudin , J. L. Davenport , K. Watanabe , T. Taniguchi , M. C. Asensio , J. Velasco , Nano Lett. 2019, 19, 2682. 30888827 10.1021/acs.nanolett.9b00649

[36] P. V. Nguyen , N. C. Teutsch , N. P. Wilson , J. Kahn , X. Xia , A. J. Graham , V. Kandyba , A. Giampietri , A. Barinov , G. C. Constantinescu , N. Yeung , N. D. M. Hine , X. Xu , D. H. Cobden , N. R. Wilson , Nature 2019, 572, 220. 31316202 10.1038/s41586-019-1402-1

[37] R. Muzzio , A. J. H. Jones , D. Curcio , D. Biswas , J. A. Miwa , P. Hofmann , K. Watanabe , T. Taniguchi , S. Singh , C. Jozwiak , E. Rotenberg , A. Bostwick , R. J. Koch , S. Ulstrup , J. Katoch , Phys. Rev. B 2020, 101, 201409.

[38] A. J. H. Jones , R. Muzzio , P. Majchrzak , S. Pakdel , D. Curcio , K. Volckaert , D. Biswas , J. Gobbo , S. Singh , J. T. Robinson , K. Watanabe , T. Taniguchi , T. K. Kim , C. Cacho , N. Lanata , J. A. Miwa , P. Hofmann , J. Katoch , S. Ulstrup , Adv. Mater. 2020, 32, 2001656. 10.1002/adma.202001656 32529706

[39] A. Kaminski , S. Rosenkranz , M. R. Norman , M. Randeria , Z. Z. Li , H. Raff , J. C. Campuzano , Phys. Rev. X 2016, 6, 031040. 10.1103/PhysRevLett.90.207003 12785917

[40] M. Naamneh , J. C. Campuzano , A. Kanigel , The electronic structure of BSCCO in the presence of a super-current: Flux-ow, doppler shift and quasiparticle pockets, 2016, arXiv:1607.02901 [cond-mat.supr-con].

[41] D. Curcio , A. J. H. Jones , R. Muzzio , K. Volckaert , D. Biswas , C. E. Sanders , P. Dudin , C. Cacho , S. Singh , K. Watanabe , T. Taniguchi , J. A. Miwa , J. Katoch , S. Ulstrup , P. Hofmann , Phys. Rev. Lett. 2020, 125, 236403. 33337178 10.1103/PhysRevLett.125.236403

[42] Y. Zhu , S. Murali , M. D. Stoller , K. J. Ganesh , W. Cai , P. J. Ferreira , A. Pirkle , R. M. Wallace , K. A. Cychosz , M. Thommes , D. Su , E. A. Stach , R. S. Ruoff , Science 2011, 332, 1537. 21566159 10.1126/science.1200770

[43] X. Li , C. W. Magnuson , A. Venugopal , R. M. Tromp , J. B. Hannon , E. M. Vogel , L. Colombo , R. S. Ruoff , J. Am. Chem. Soc. 2011, 133, 2816. 21309560 10.1021/ja109793s

[44] A. K. Patra , S. Singh , B. Barin , Y. Lee , J.-H. Ahn , E. del Barco , E. R. Mucciolo , B. Ozyilmaz , Appl. Phys. Lett. 2012, 101, 162407.

[45] S. Ulstrup , R. J. Koch , D. Schwarz , K. M. McCreary , B. T. Jonker , S. Singh , A. Bostwick , E. Rotenberg , C. Jozwiak , J. Katoch , Appl. Phys. Lett. 2019, 114, 151601.

[46] T. Ohta , J. T. Robinson , P. J. Feibelman , A. Bostwick , E. Rotenberg , T. E. Beechem , Phys. Rev. Lett. 2012, 109, 186807. 23215315 10.1103/PhysRevLett.109.186807

[47] I. Razado-Colambo , J. Avila , J. P. Nys , C. Chen , X. Wallart , M. C. Asensio , D. Vignaud , Sci. Rep. 2016, 6, 27261. 27264791 10.1038/srep27261 PMC4893698

[48] H. Peng , N. B. M. Schroter , J. Yin , H. Wang , T.-F. Chung , H. Yang , S. Ekahana , Z. Liu , J. Jiang , L. Yang , T. Zhang , C. Chen , H. Ni , A. Barinov , Y. P. Chen , Z. Liu , H. Peng , Y. Chen , Adv. Mater. 2017, 29, 1606741. 10.1002/adma.201606741 28481053

[49] J. J. P. Thompson , D. Pei , H. Peng , H. Wang , N. Channa , H. L. Peng , A. Barinov , N. B. M. Schroter , Y. Chen , M. Mucha-Kruczyński , Nat. Commun. 2020, 11, 3582. 32681042 10.1038/s41467-020-17412-0 PMC7367817

[50] I. A. Nechaev , M. F. Jensen , E. D. L. Rienks , V. M. Silkin , P. M. Echenique , E. V. Chulkov , P. Hofmann , Phys. Rev. B 2009, 80, 113402.

[51] I. Gierz , J. Henk , H. Hochst , C. R. Ast , K. Kern , Phys. Rev. B 2011, 83, 121408.

[52] A. V. Kretinin , Y. Cao , J. S. Tu , G. L. Yu , R. Jalil , K. S. Novoselov , S. J. Haigh , A. Gholinia , A. Mishchenko , M. Lozada , T. Georgiou , C. R. Woods , F. Withers , P. Blake , G. Eda , A. Wirsig , C. Hucho , K. Watanabe , T. Taniguchi , A. K. Geim , R. V. Gorbachev , Nano Lett. 2014, 14, 3270. 24844319 10.1021/nl5006542

[53] T.-F. Chung , Y. Xu , Y. P. Chen , Phys. Rev. B 2018, 98, 035425.

[54] H. Polshyn , M. Yankowitz , S. Chen , Y. Zhang , K. Watanabe , T. Taniguchi , C. R. Dean , A. F. Young , Nat. Phys. 2019, 15, 1011. 10.1126/science.aav1910 30679385

